# The effect of vitrification after warming on the expressions of p38, CDK1, and cyclin B in immature goat oocytes followed by *in vitro* maturation

**DOI:** 10.14202/vetworld.2020.2126-2132

**Published:** 2020-10-10

**Authors:** A. A. Muhammad Nur Kasman, Budi Santoso, Widjiati Widjiati

**Affiliations:** 1 Student of Doctoral Program Medicine Science, Faculty of Medicine, Universitas Airlangga, Surabaya, Indonesia; 2 Faculty of Health Science, Universitas Muhammadiyah Mataram, Mataram, Indonesia; 3 Department of Obstetrics and Gynecology, Faculty of Medicine, Universitas Airlangga, Surabaya, Indonesia; 4 Department of Veterinary Anatomy, Faculty of Veterinary Medicine, Universitas Airlangga, Surabaya, Indonesia

**Keywords:** CDK1, cyclin B, *in vitro* maturation, oocytes, p38, vitrification

## Abstract

**Background and Aim:**

The combination of vitrification techniques and *in vitro* maturation can reduce oocyte competence. Mitogen-activated protein kinase and maturation-promoting factor are significant in oocyte meiotic maturation regulation. This study aimed to analyze vitrification’s effect, after warming followed by *in vitro* maturation, on the expressions of protein 38 (p38), cyclin-dependent kinase 1 (CDK1), and cyclin B and oocyte maturation level.

**Materials and Methods:**

Immature goat oocytes were soaked in vitrification and warming solutions. The procedure was followed by *in vitro* maturation and *in vitro* maturation without post-warming vitrification as a control. These oocytes, along with their cumulus, were vitrified using *hemistraw* in liquid nitrogen. Oocyte maturation was carried out in a maturation medium that was added with 10 μg/mL of FSH, 10 μg/mL of LH, and 1 μg/mL E_2_ for 22 h. The expressions of p38, CDK1, and cyclin B were observed using immunocytochemical methods, which were assessed semiquantitatively according to the modified Remmele method. The oocyte maturation level was observed using the aceto-orcein staining method based on the achievement of chromosomes up to the metaphase II stage and/or the formation of the polar body I.

**Results:**

p38 expression in vitrified oocytes after warming, followed by *in vitro* maturation, increased insignificantly (p≥0.05), with the acquisition of 3.91±2.69 and 2.69±0.50 in the control oocytes. CDK1 expression in vitrified oocytes decreased significantly (p≤0.05) after warming, followed by *in vitro* maturation, with the acquisition of 2.73±1.24 and 7.27±4.39 in the control oocytes. Cyclin B expression in vitrified oocytes decreased insignificantly (p≥0.05) after warming, followed by *in vitro* maturation, with the acquisition of 3.09±1.4 and 4.18±2.61 in the control oocytes. The proportion of vitrified oocyte maturation levels after warming, followed by *in vitro* maturation, decreased significantly (p≤0.05), with the acquisition of 45.45% and 77.27% in the control oocytes.

**Conclusion:**

This study concluded that vitrification after warming resulted in an insignificant increase in p38 expression, a significant decrease in CDK1 expression, an insignificant decrease in cyclin B expression, and a significant reduction in oocyte maturation levels.

## Introduction

The current application of a combination of vitrification techniques and oocyte maturation is often carried out in terms of methods, oocyte storage time, and their effect on oocyte competence [1]. The combination of vitrification techniques and *in vitro* maturation can still reduce oocyte competence [2]. Vitrification, a cryopreservation method at high temperatures, uses small volumes and high cryoprotectant concentrations [3]. Some researchers report that oocyte vitrification can cause changes in protein maturation and expression [4], oocyte ultrastructure [5], gene expression [6,7], and deoxyribonucleic acid [8]. Rapid temperature changes during vitrification, accompanied by the use of high cryoprotectant concentrations, can cause osmotic stress, which results in loss of oocyte defense mechanisms [9].

Mitogen-activated protein kinase (MAPK) and maturation-promoting factor (MPF) are significant in the regulation of oocyte meiotic maturation [10]. Oocyte vitrification can suppress the activation of the MAPK pathway [11]. Protein 38 (p38) is a group of MAPKs that are responsive to stress stimuli, such as cytokines, heat, and osmotic changes involved in cell differentiation [12]. Oocyte maturation involves the activation of various signal transduction pathways to activate the MPF, consisting of catalytic subunits, namely, cyclin-dependent kinase 1 (CDK1) and regulator subunit cyclin B [13,14], which are good markers for oocyte development [15]. MPF activity requires the CDK1 and cyclin B subunit complexes through CDK1 phosphorylation at Thr161 by a cdc2-activating kinase (CAK) and dephosphorylation at threonine 14 (Thr14) and tyrosine 15 (Tyr15) by Cdc25 phosphatase [16,17].

There is limited scientific information on the changes in the regulation of p38 expression with CDK1 and cyclin B as well as the level of oocyte maturation as a result of vitrification after warming, followed by *in vitro* maturation. In this study, we analyzed the effect of vitrification after warming, followed by *in vitro* maturation on the expressions of p38, CDK1, and cyclin B and oocyte maturation levels.

## Materials and Methods

### Ethical approval

The ethical clearance certificate number 2.KE.058.04.2019 was obtained from the Faculty of Veterinary Medicine Universitas Airlangga, Surabaya.

### Study period and location

This experimental study was carried out at the Faculty of Veterinary Medicine, Airlangga University, Surabaya, from June 17, 2019, to August 23, 2019.

### Materials

The cumulus-oocyte complex (COC) was obtained from goat ovaries aged 6 months to 1.5 years with cumulus of at least three layers. The COC complex was aspirated on the surface of the ovarian follicle with a diameter of 2–6 mm and vitrified, and then, warming, followed by *in vitro* maturation was carried out.

The study materials included the following: Liquid nitrogen, physiological NaCl (0.9%), penicillin G (75 μg/mL), streptomycin sulfate (50 μg/mL), phosphate-buffered saline (PBS) (pH 7.4), vitrification solution (Cryotech Lab, Japan), warming solution (Cryotech Lab, Japan), mineral oil (Vitrolife, Sweden), G-MOP PLUS (Vitrolife^®^), 10 μg/mL of FSH, 10 μg/mL of LH, 1 μg/mL of E_2_, p38 antibody (Santa Cruz Biotechnology, USA), CDK1 antibody (Cloud-Clone, USA), cyclin B antibody (Cloud-Clone, USA), 1% hyaluronidase enzyme (Vitrolife, Sweden), 1% aceto-orcein, and protein expression examination with immunocytochemistry (Thermo Fisher Scientific, USA).

The equipment used in this study were 35- and 65-mm Petri dishes (Thermo Fisher Scientific, USA), glass object, cover glasses, disposable syringes (1, 3, and 10 mL), 18-G needles, 0.25-mL *hemistraw*, micro sterilizer 0.2 μm (Thermo Fisher Scientific, USA), liquid nitrogen container, thermos, water bath, sterile chamber, glass beaker, tweezers, scissors, Pasteur pipette (Merck, Germany), Eppendorf micropipette, refrigerator, CO_2_ incubator (Thermo Fisher Scientific, USA), inverted microscope (Meiji Techno, Japan), and CX41 microscope (Olympus, Japan).

### Methods

#### Oocyte collection

The ovaries came from slaughterhouses to the laboratory using a thermos. They were put into a glass beaker containing 0.9% physiological NaCl (0.9%) with penicillin G (75 μg/mL) and streptomycin sulfate (50 μg/mL) at 37°C. The COC was aspirated in the follicles with a diameter of 2-6 mm by thrusting a 10-mL disposable syringe with an 18-G needle containing PBS. All aspirations were put into a Petri dish for evaluation under a 40× magnified, inverted microscope. After evaluation, the COC with intact cytoplasm and cumulus cells of at least three layers were selected and washed with PBS 3 times.

#### Vitrification and warming

The collected oocytes were exposed to the vitrification solution (Cryotech Lab, Japan) in stages, equilibrium solution for 12-15 min, vitrification solution 2 for 30-30 s, and vitrification solution 3 for 10-20 s. Next, the oocytes were put into 0.25-mL transparent *hemistraw* and liquid nitrogen for 7 days. After 7 days of exposure, these were diluted gradually in warming solution (Cryotech Lab, Japan), thawing solution for 1 min, diluent solution for 3 min, warming solution 1 for 5 min, and warming solution 2 for 1 min.

### *In vitro* maturation

Oocyte maturation was carried out in a maturation medium that was added with 10 μg/mL of FSH, 10 μg/mL of LH, and 1 μg/mL E_2_ for 22 h. Furthermore, drops were made on a 35-mm disposable Petri dish (1 drop = 50 μL) (each drop contained ±3-4 oocytes) and then fixed with mineral oil (Cryotech Lab, Japan). Petri dishes were placed in an incubator with environmental conditions of 5% CO_2_, temperature of 37.5°C, and maximum humidity level (95-99%) for 22 h. Subsequently, observations were made on each treatment using an CX41 microscope (Olympus, Japan).

### Immunocytochemistry

The oocytes were put on a glass object coated with poly-L-lysine and then covered with a cover glass. Fixation of oocytes was conducted by putting the glass object into a container with acetic acid (glacial) 100% and ethanol absolute with a ratio of 1:3 for a minimum of 24 h before immunocytochemical coloring was performed. The fixated oocyte preparation was dropped with hydrogen peroxide 3% for 5-10 min, washed with PBS (pH 7.4) 2 times for 5 min, and then added with trypsin for 15 min in (0.025%) the incubator with a temperature of 37°C. Subsequently, we washed it with PBS 2 times for 5 min, added with Ultra V Block for 5 min, rinsed with PBS for 5 min, added with the antibody for 60 min at 27°C temperature added with biotinylated link (yellow) drops for 30 min, and washed with PBS 2 times for 5 min. It was then added with streptavidin (red) drops for 30 min at room temperature. Next, it was washed with PBS 2 times for 5 min. Afterward, the chromogen 3,3′-diaminobenzidine tetrahydrochloride was added for 6-10 min. It was then washed with PBS 2 times for 5 min and rinsed with aqua dest for 5 min. The last step was adding methylene green for 5-10 min. Finally, the coloring remains were slowly absorbed with a paper towel until the water was significantly reduced.

#### Observation of protein expression

The p38, CDK1, and cyclin B expressions were observed using an CX41 microscope (Olympus, Japan). The results of each expression were assessed semiquantitatively according to the modified Remmele method [18]. The index Remmele scale was the result of multiplying the percentage score of immunoreactive cells with the color intensity scores produced on the cell, as shown in Table-1.

**Table 1:** Semi-quantitative scale index Remele scale is the result of multiplying the positive cell percentage score (a) with the color reaction intensity score (b), so the IRS scale=(a×b).

(a)	(b)
Score 0: No positive cells	Score 0: No color reaction
Score 1: Positive cells <10%	Score 1: Low color intensity
Score 2: Positive cells between 11% and 50%	Score 2: Medium color intensity
Score 3: Positive cells between 51% and 80%	Score 3: Strong color intensity
Score 4: Positive cells over than 80%	

### Observation of the oocyte maturation level

We observed the oocyte maturation level using an CX41 microscope (Olympus, Japan). Data on the maturity level of oocytes classified as mature were determined based on the achievement of chromosomes up to the metaphase II stage and/or the formation of the polar body I by the aceto-orcein staining method. Oocytes resulting from vitrification and *in vitro* maturation were included in PBS, which were given 1% hyaluronidase for some time, while repeated pipetting was performed. The oocytes were removed from Petri dishes containing PBS, and their cumulus cells were released by repetitive pipetting, so they became bald, and we placed them on the glass object. Then, they were covered with a cover glass and fixed in a solution of acetic acid–ethanol (1:3) for 48 h. The oocytes were stained with 1% aceto-orcein dye through one of the cover glasses and allowed to stand for 10 min. Then, they were rinsed with a destaining solution, while a tissue paper was used to absorb the liquid and consequently dry the preparation. Furthermore, the preparations were observed using a microscope.

### Statistical analysis

Data were analyzed using the SPSS 24.0 software (IBM Corp., NY, USA) and first tested for normality using the Kolmogorov–Smirnov test. Furthermore, nonparametric data were verified using the Mann–Whitney tests, and p≤0.05 was considered statistically significant.

## Results and Discussion

### p38 expression

**Figure-1:**
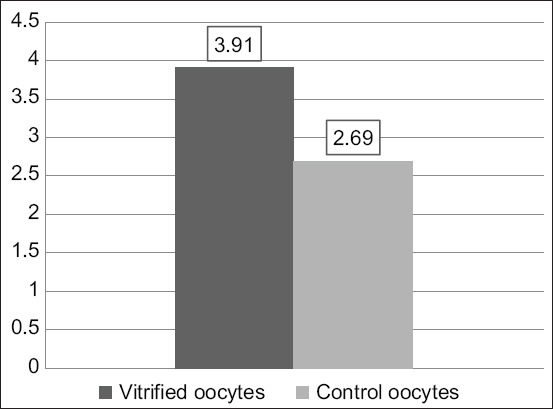
Histogram of vitrified p38 oocyte expression after warming continued by *in vitro* maturation and control oocytes.

**Figure-2:**
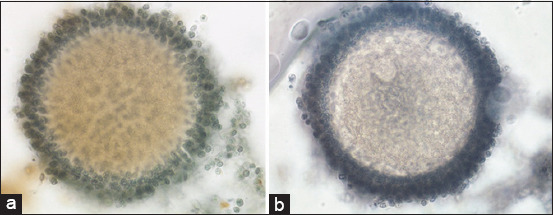
Expression of vitrified p38 oocytes after warming followed by *in vitro* maturation with a magnification of 400× (a), control oocytes (b).

The vitrification–warming treatment over time stepped oocytes, followed by *in vitro* maturation with the addition of 10 μg/mL of FSH, 10 μg/mL of LH, and 1 μg/mL of E_2_ resulted in an insignificant increase in the p38 expression (3.91±2.69 vs. 2.69±0.50; p≥0.05; Table-2, Figure-1). The expression of p38 in vitrified oocytes after warming was followed by *in vitro* maturation (Figure-2). The vitrified oocytes (a) showed higher p38 expression than the control oocytes (b).

**Table 2:** Mean values±standard deviations of the p38 expression.

Protein expression (n=22)	Vitrified oocytes	Control oocytes	p-value
p38	3.91±2.69	2.69±0.50	0.428

*Significant at α≤0.05

### CDK1 expression

**Figure-3:**
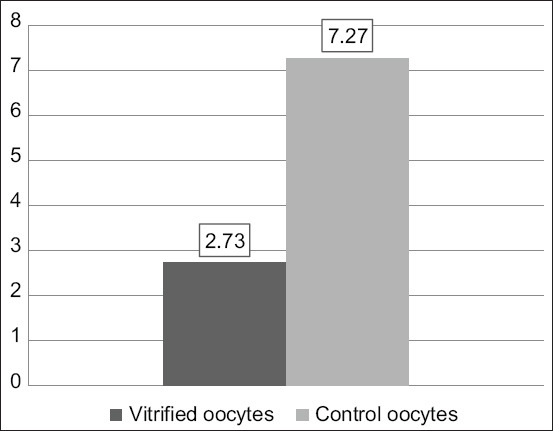
Histogram of vitrified oocyte CDK1 expression after warming continued by *in vitro* maturation and control oocytes.

**Figure-4:**
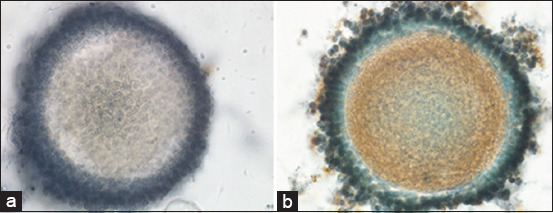
CDK1 expression of vitrified oocytes after warming followed by *in vitro* maturation with a magnification of 400× (a), control oocytes (b).

The vitrification–warming treatment over time stepped oocytes, followed by *in vitro* maturation with the addition of 10 μg/mL of FSH, 10 μg/mL of LH, and 1 μg/mL of E_2_ resulted in a significant reduction in the CDK1 expression (2.73±1.24 vs. 7.27±4.39; p≤0.05; Table-3, Figure-3). The expression of CDK1 in vitrified oocytes after warming was followed by *in vitro* maturation (Figure-4). The vitrified oocytes (a) showed lower CDK1 expression than the control oocytes (b).

**Table 3:** Mean values±standard deviations of the CDK1 expression.

Protein expression (n=22)	Vitrified oocytes	Control oocytes	p-value
CDK1	2.73±1.24	7.27±4.39	0.019*

* Significant at α≤0.05

### Cyclin B expression

**Figure-5:**
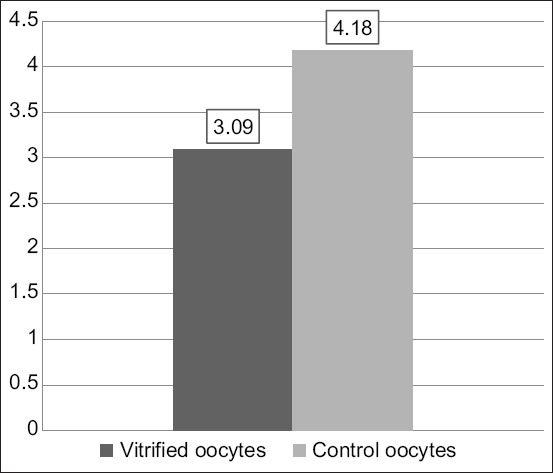
Histogram of expression of Cyclin B oocyte vitrification after warming continued by *in vitro* maturation and control oocytes.

**Figure-6:**
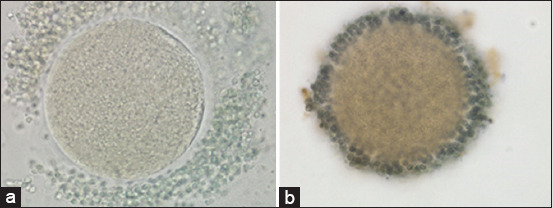
Cyclin B expression of vitrified oocytes after warming followed by *in vitro* maturation with a magnification of 400× (a), control oocytes (b).

The vitrification–warming treatment over time stepped oocytes, followed by *in vitro* maturation with the addition of 10 μg/mL of FSH, 10 μg/mL of LH, and 1 μg/mL of E_2_, resulted in an insignificant reduction in the cyclin B expression (3.09±.4 vs. 4.18±2.61; p≥0.05; Table-4, Figure-5). The expression of cyclin B in vitrified oocytes after warming was followed by *in vitro* maturation (Figure-6). The vitrified oocytes (a) showed lower cyclin B expression than the control oocytes (b).

**Table 4:** Mean values ± standard deviations of the Cyclin B expression.

Protein expression (n=22)	Vitrified oocytes	Control oocytes	p-value
Cyclin B	3.09±1.4	4.18±2.61	0.107

*Significant at α≤0.05

### Oocyte maturation level

**Figure-7:**
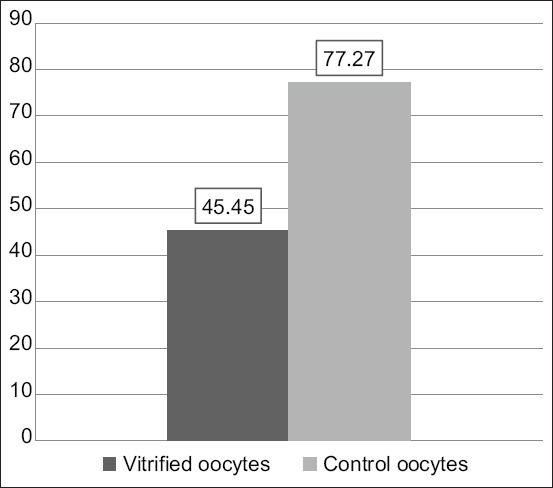
Histogram of vitrified oocyte maturation level after warming continued by *in vitro* maturation and control oocytes.

The vitrification–warming treatment over time stepped oocytes, which followed by *in vitro* maturation with the addition of 10 μg/mL of FSH, 10 μg/mL of LH, and 1 μg/mL of E_2_, resulted in a significant reduction in oocyte maturation level (45.45 vs. 77.27; p≤0.05; Table-5, Figure-7).

**Table 5:** Proportion of mature oocytes.

Variable (n=22)	Vitrified oocytes	Control oocytes	p-value
Mature oocytes (%)	45.45	77.27	0.032*

* Significant at α≤0.05

Oocyte maturation is characterized by changes in the cell nucleus morphology, namely, changes in the oocytes from the diplotene stage to the metaphase II stage so that they are ready to be fertilized. These changes begin with the movement of the cortex granules into the periphery. Then, the nucleus membrane fuses with the nucleus and enlarges and then fuses, in which then the oocyte nucleus divides in meiosis to the metaphase II stage. Oocyte maturation is controlled by the activation of the signal transduction pathway through the MAPK and MPF regulatory pathways. MAPK is responsible for initiating the GVBD stage of oocyte meiosis to maintain the oocyte stage during metaphase II [19,20]. MPF regulates the meiotic cell cycle continuity from the diplotene stage to the metaphase II stage, which affects oocyte competence to reach the next developmental stage [21].

Our study found that the expression of MPF (CDK1 and cyclin B) in vitrified oocytes after warming followed by *in vitro* maturation decreased compared with that in the control oocytes. This proves that vitrification treatment can decrease MPF expression, which consists of CDK1 catalytic and cyclin B regulator subunits, one of the leading causes of low oocyte competence. This finding might be due to toxic induction during vitrification with the use of high cryoprotectant concentrations, which affect changes in phosphorylation and dephosphorylation of proteins in oocytes, reducing their potential to sustain metabolic damage during vitrification.

MPF activity requires the CDK1 and cyclin B subunit complexes through phosphorylation of CDK1 on Thr161 by CAK and dephosphorylation on Thr14 and Tyr15 by Cdc25 phosphatase [22]. The p38MAPK phosphorylation activity in serine 309 activates Cdc25 phosphatase, which allows the binding of 14-3-3 proteins, resulting in Cdc25B inhibition [23]. Other researchers state that MPF activation is also regulated by a balance of Wee1/Myt1 kinase regulator activity, which inhibits CDK1 dephosphorylation on Thr14 and Tyr15 and Cdc25, activating CDK1 dephosphorylation at the same site [24]. Both of these kinase inhibitors (Wee1 and Myt1) can phosphorylate CDK1 on Tyr15, but only Myt1 can also phosphorylate on Thr14 [25]. High Cdc25 activity and low Wee1/Myt1 are needed to activate CDK1. When combining cyclin B with CDK1, the oocyte cycle is at a transition stage between G2 and M in the nucleus and becomes active again when the oocyte begins the meiotic division stage with the inactivation of the Wee1/Myt1 inhibitor. The transition from G2 to M then occurs through the activation of CDK1 and cyclin B as a result of dephosphorylation of Thr14 and Tyr15 by a protein phosphatase called Cdc25 phosphatase [26,27]. Thus, the disruption of the regulation of Cdc25 phosphatase, CAK, Wee1/Myt1, and p38MAPK during vitrification treatment followed by *in vitro* maturation causes a decrease in the expressions of CDK1 and cyclin B, which affects oocyte competence to mature.

This study indicated that the p38MAPK expression in vitrified oocytes after warming, followed by *in vitro* maturation, increased insignificantly compared with that in the control oocytes. Vitrification treatment can cause an increase in p38MAPK expression. Decreased p38MAPK expression is possible due to an increase in ROS that is mediated by an increase in Ca^2+^ oscillations in oocytes during vitrification, followed by warming. Vitrification will trigger ROS formation, which can cause the accumulation of Ca^2+^ in oocytes stored in the endoplasmic reticulum through the mediator inositol 1,4,5-trisphosphate [28,29]. Ca^2+^ ions are significant messengers in responding to intracellular environmental stressors, which induce increased Ca^2+^ concentrations into the cytoplasm. Vitrification can interfere with the Ca^2+^ cycle, which can limit oocyte development [30]. Cryoprotectant agents are commonly used when cryopreservation has been shown to induce significant changes in intracellular Ca^2+^ concentrations [31]. The results of another study reported that oocytes after vitrification continued warming showed a decrease in the potential of Ca^2^^+^ during oocyte activation [32].

The p38MAPK member regulator contributes to the cellular mechanism of osmotic stress responses, including the regulation of intracellular levels of organic and inorganic ions in cells [33]. Osmotic stress occurs due to differences between intracellular and extracellular osmolalities, which induces swelling and shrinkage of cells as a consequence of the entry of water that can disrupt cell activity. Researchers suspect that increased p38MAPK expression in vitrified oocytes after warming can be caused by an increase in ROS mediated by HSP70. The p38MAPK protein is a member of the MAPK group that is responsive to stress stimuli, such as cytokines, heat, and osmotic changes involved in cell differentiation [34]. The p38MAPK protein identified as 38-kDa polypeptide will undergo tyrosine phosphorylation as an adaptive and physiological response from endotoxin treatment and hyperosmolarity shock, which is obtained during changes in the physical and chemical environment in oocyte medium after vitrification, such as changes in nutrient concentration, growth factors, and cytokines [35]. Signaling complexes, mediated by other protein interactions, regulate p38MAPK proteins, which have implications for MAPK functional regulation. In this case, there may be a joint functional regulation between p38 and HSP70 in regulating the environmental stress vitrification effects, as reported by other researchers [36]. HSP70 is a potential chaperone for translational p38 *in vivo* and *in vitro* due to environmental stress through phosphorylation of MAPK-activated protein kinase (MK2) [37]. Regarding HSP70, other researchers report that the influence of osmotic stress on vitrified oocytes after warming causes an increase in HSP70 expression [38].

## Conclusion

This study concluded that oocyte vitrification after warming followed by *in vitro* maturation caused an insignificant increase in p38 expression, a significant decrease in CDK1 expression, an insignificant decrease in cyclin B expression, and a significant reduction in oocyte maturation levels.

## Authors’ Contributions

AAMNK, BS, and WW designed this research. AAMNK conducted a survey and took samples at the samples field. All authors examined samples in the research laboratory. All authors compiled, read, revised, and approved the final manuscript.
